# Clinical outcomes of teeth adjacent to the site of mandibulotomy or mandibulectomy in patients with head and neck cancer: results from a multidisciplinary mono-institutional head and neck tumor board

**DOI:** 10.1186/s12903-023-03050-7

**Published:** 2023-06-03

**Authors:** Raffaella Castagnola, Cosimo Rupe, Gioele Gioco, Giovanni Almadori, Jacopo Galli, Luca Tagliaferri, Alessandra Cassano, Patrizia Gallenzi, Carlo Lajolo

**Affiliations:** 1 Head and Neck Department, School of Dentistry, Fondazione Policlinico Universitario A. Gemelli-IRCCS, Università Cattolica del Sacro Cuore, Largo A. Gemelli, 8, Rome, 00168 Italy; 2 Head and Neck Department, Institute of Otolaryngology, Fondazione Policlinico Universitario A. Gemelli-IRCCS, Università Cattolica del Sacro Cuore, Largo A. Gemelli, 8, Rome, 00168 Italy; 3 Department of Radiation Oncology, Institute of Radiology, Fondazione Policlinico Universitario A. Gemelli- IRCCS, Università Cattolica del Sacro Cuore, Largo A. Gemelli, 8, Rome, 00168 Italy; 4 Department of Medical Oncology, Institute of Radiology, Fondazione Policlinico Universitario A. Gemelli- IRCCS, Università Cattolica del Sacro Cuore, Largo A. Gemelli, 8, Rome, 00168 Italy

**Keywords:** Head and Neck Cancer, Oral Cancer, Mandibulotomy, Mandibulectomy, Tooth prognosis, Tooth survival

## Abstract

**Introduction:**

The aim of this case series was to evaluate the necrosis of teeth adjacent to the site of mandibulotomy or mandibulectomy in a cohort of patients suffering from head and neck cancers.

**Methods:**

Fourteen patients who underwent segmental mandibulectomy or paramedian mandibulotomy for oral, oropharynx or major salivary gland cancer and a total of 23 teeth were included in this case series. Twelve patients underwent adjuvant head and neck radiotherapy. Cold sensitivity pulp testing and/or electric pulp testing were performed on teeth at the margin of mandibulectomy and on teeth adjacent to mandibulotomy after surgery. A “positive” response was considered the healthy state, and “negative” was considered the diseased state of the tooth.

**Results:**

The 10 patients who underwent mandibulotomy had 12 teeth with a negative response. The 4 patients treated by mandibulectomy had two positive and three negative responses to cold and electric pulp tests. Fifteen out of 23 teeth (65.2%) showed a negative response to sensitivity testing.

**Conclusions:**

Tooth necrosis seems to be a common event after mandibulectomy and mandibulotomy.

**Clinical Relevance:**

To avoid post-surgery complications, performing root canal therapy before surgery on the teeth adjacent to the surgical site could be an appropriate strategy.

## Introduction

Head and neck cancers (HNC) are the seventh most common neoplasia worldwide: every year, more than 900,000 new cases of HNC are diagnosed [1]. Oral cancers represent approximately 40% of all HNC, with 377,713 new diagnoses [2, 3].

Surgery, alone or in combination with radiotherapy (RT) and/or Chemotherapy (CT), is the most common initial definitive treatment for the majority of oral cancers [4]. In the case of the mandible, two different surgical resections can be performed. In the past, it was thought that the spread of cancer could occur via the lymphatic system within the mandibular periosteum; thus, removal of the cancer was performed with segmental mandibulectomy, causing aesthetic and functional problems [5, 6]. Subsequently, authors have shown that some cancers directly invade the jaws but not the mandibular cortex, allowing partial thickness (marginal) mandibulectomy to be performed in some cases [7]. Marginal mandibulectomy is a technique that consists of the removal of the alveolar bone while maintaining the inferior border of the mandible. These techniques for oral cancer removal can weaken the mandible structure; thus, a reconstruction plate is often required to avoid mandible fracture [8].

In the presence of deep tumours in the posterior oropharynx and oral cavity, access to the tumours is usually achieved through mandibulotomy [7]. In 1839, this osteotomy technique was introduced, and it was then modified in the design of osteotomy (straight, notched, or stair-stepped), in the site (midline, paramedian) and in the fixation method [9]. Several postoperative complications were reported, including extraction of the teeth adjacent to the mandibulotomy site and inferior alveolar nerve injury [10].

In recent decades, many studies have tried to define the fate of HNC patients’ teeth, especially those remaining anterior or posterior to a segmental resection of the mandible [11–13], without reaching certain results. Furthermore, the fate of teeth located at the margins of the mandibulotomy has not yet been defined. In particular, it is unclear whether surgical resection may directly or indirectly cause the loss of vitality of teeth located at the edges of mandibulectomy and/or adjacent to mandibulotomy. The aim of this case series was to evaluate whether tooth necrosis is a common event following mandibulectomy or mandibulotomy in a cohort of patients suffering from HNC.

## Materials and methods

Fourteen patients suffering from HNC seeking treatment at the Oral Medicine, Head and Neck Department, Fondazione Policlinico Universitario A. Gemelli-IRCSS between July 2017 and August 2022 were consecutively recruited for this study. This case series represents a subgroup analysis of a wider prospective cohort study, which was registered on ClinicalTrials.gov (ID: NCT04009161) and was approved by the ethical committee of the ‘Fondazione Policlinico Universitario A. Gemelli IRCCS in Rome’ (22,858/18). The study was conducted in accordance with the Declaration of Helsinki, and all patients signed an informed consent form. After major oncologic surgery, a multidisciplinary dental team usually checks the condition of the oral cavity and dental health to treat and prevent further dental and oral diseases. Special attention is needed when further RT treatment is needed, considering the higher risk of oral sequelae. All patients are visited with the support of orthopantomography (OPT), and periapical radiographs, whenever possible, are performed to detect dental diseases [14, 15]. First, anagraphic and anamnestic data were carefully recorded, particularly focusing on the oncologic history of the patient and on exposure to risk factors for oncologic and dental diseases. The following variables were recorded: sex, age, previous or scheduled oncological treatment (RT and surgery), and site of the tumour.

### Subject population

**Fig. 1:**
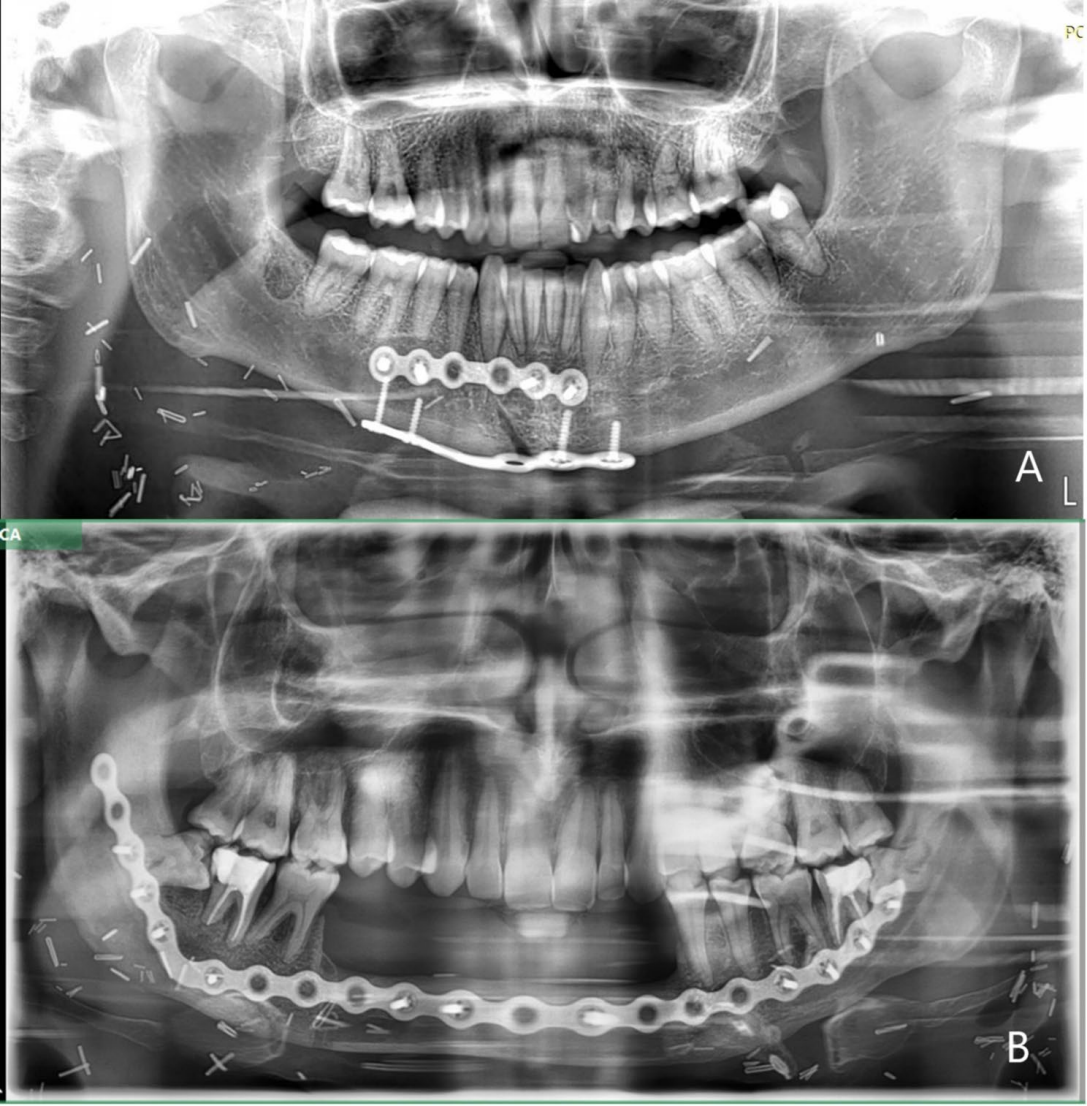
Panoramic radiographs of patients who underwent right paramedian mandibulotomy (tongue SCC) (**A**), and left and right segmental mandibulectomy (Floor of the mouth and tongue SCC) (**B**)

Fourteen patients who underwent segmental mandibulectomy (Fig. 1B) or paramedian mandibulotomy (Fig. 1A) between July 2017 and March 2022 for oral, oropharynx or major salivary gland cancer were included in this case series and observed after surgery (Table 1). Eight patients were affected by tongue squamous cell carcinoma (SSC), 1 by oropharynx SCC, 3 by floor of the mouth SCC, 1 by retromolar trigon SCC, and 1 by adenocarcinoma of the parotid gland. There were 10 males and 4 females with an average age of 55 years (27–79 years). Twelve patients underwent adjuvant head and neck RT.

**Table 1:** Characteristics of the included patients and teeth

Patient	Age	S	Tumour site	Surgery	Timing Sur-Vis (days)	Timing Vis-Trt (days)	Follow-up^*^	Marginal teeth	Sensitivity testing	Trt	RT
F.D (1)	27	M	Floor of the mouth and tongue	Left and right segmental mandibulectomy	621	- -	5 years	4.6 3.5	Positive Positive	- -	Yes
G.R. (2)	58	M	Floor of the mouth and tongue	Right paramedian mandibulotomy	54	959	5 years	3.3	Negative	Endo	Yes
P.M. (3)	70	F	Parotid	Left paramedian mandibulotomy	60	268 268	3 years	3.3 3.2	Negative Negative	Endo Endo	Yes
S.R. (4)	54	M	Tongue	Left paramedian mandibulotomy	114	7 -	5 months	3.3 3.2	Negative Positive	Ext -	No
P.R. (5)	51	M	Tongue	Left paramedian mandibulotomy	64	- -	17 days	3.3 3.2	Positive Positive	- -	No
S.R. (6)	67	M	Retromolar trigone	Left segmental mandibulectomy	14	-	2 years	3.3	Negative	-	No
O.A. (7)	51	M	Tongue	Left paramedian mandibulotomy	26	21 21	2 years	3.2 3.3	Negative Negative	Ext Ext	Yes
G.C. (8)	59	M	Floor of the mouth, tongue, mandible	Left and right segmental mandibulectomy	50	-	1 year	3.5	Negative	-	Yes
D. A. (9)	50	M	Oropharynx	Right paramedian mandibulotomy	18	- -	2 months	4.3 4.2	Positive Positive	- -	Yes
S.S. (10)	56	F	Tongue	Right paramedian mandibulotomy	79	99	1 year	4.3	Negative	Endo	Yes
D.G. (11)	54	F	Tongue	Right paramedian mandibulotomy	228	345 345	7 years	4.1 3.1	Negative Negative	Endo Endo	No
K. J. (12)	32	F	Tongue	Right paramedian mandibulotomy	51	- -	1 year	4.2 4.3	Positive Negative	- -	Yes
P. A. (13)	68	M	Tongue	Right paramedian mandibulotomy	58	- -	18 days	4.3 4.2	Negative Negative	- -	Yes
T. F. (14)	65	M	Tongue	Right segmental mandibulectomy	64	-	1 month	4.1	Negative	-	Yes

Age: age of the patient; S: sex; Tumor site: tumor localization; Surgery: kind of surgery used; Timing Sur-Vis: temporal distance between surgery and dental visit; Timing Vis-Trt: temporal distance between dental visit and endodontic treatment or tooth extraction; Marginal Teeth: marginal teeth at mandibulotomy or mandibulectomy, Sensitivity testing: response of cold and/or electric testing; Trt: Treatment; RT: patient who underwent head and neck radiotherapy, Endo: endodontic treatment, Ext: tooth extraction. * Follow-up is rounded up to the nearest month or year

Dental visits were performed in a period ranging from 14 to 621 days after surgery. Cold sensitivity pulp testing was performed on teeth at the margin of mandibulectomy and on teeth adjacent to mandibulotomy. “Positive” response was considered as the healthy state and “negative” as diseased state of the tooth [16]. When the sensitivity cold testing was uncertain or negative, teeth underwent sensitivity electric pulp testing (Digitest 3, Parkell, Inc., Edgewood, NY).

Overall, 23 teeth were evaluated: 10 canines, 10 lower incisors, 2 premolars and 1 molar.

### Sensitivity pulp testing

#### Cold testing

A #2 cotton pellet was sprayed directly with cold spray (Prodonto, Hager Werken, Duisburg, Germany) from a distance of 5 mm and a period of 3 s. The cotton pellet was placed in the midface of the teeth for 10 s on both the marginal teeth of mandibulotomy or mandibulectomy as well as the adjacent teeth [17]. The patient response was recorded.

#### Electric pulp testing

When cold testing had a negative response, electric pulp testing was used according to the manufacturer’s instructions. The clip was placed on the lips, and then the tip of the tester was located on the tooth surface. A gel was placed on the tooth to help the instrument conduct electricity to obtain a more precise measurement. Then, we started from 0 on the screen and then increased until a response from the patient was reached (or no response in case of a negative result).

### Treatment

Even in the case of a negative sensitivity test, the teeth were initially left untreated unless they originated dental abscesses, visible radiographic radiolucency and/or painful response to percussion.

## Results

The results are shown in Table 1. The 10 patients who underwent mandibulotomy had 12 teeth with negative responses and 6 with positive responses, while the 4 patients treated by mandibulectomy had two positive and three negative responses on teeth using cold and electric pulp testing.

Although the teeth were initially left untreated, nine of them needed treatment during the follow-up period: six teeth were endodontically treated, while three teeth were extracted. All the extracted teeth were negative at the sensitivity testing. The mean follow-up of these patients (after the pulp sensitivity evaluation) was more than two years (735 days, range: 17-2664 days). Vital teeth received a mean follow-up of more than one year (426 days).

## Discussion

HNC treatment is complex and currently requires a multidisciplinary approach, including several specialists (i.e., head and neck surgeons, radiation oncologists, medical oncologists, oral oncologists, and maxillofacial surgeons) [18].

Surgery is the first-line therapeutic option for treating oral cancer. Segmental and marginal mandibulectomy are different techniques to resect cancer and avoid disease progression [5]. Mandibulotomy is a procedure that aims to allow better access for tumour ablative surgery. Although these procedures have two different objectives, they all involve dental elements, either at the margin of mandibulectomy or adjacent to the osteotomy.

Necrosis of a dental element can lead to apical periodontitis and dental abscess. In patients who undergo mandibulotomy and mandibulectomy, these tooth conditions should be avoided, especially in teeth adjacent to the site of mandibulotomy or mandibulectomy, to promote the healing of the surgical site or maintain its integrity. Moreover, it has been reported that mandibulotomy predisposes patients to developing ORN at the mandibulotomy site [10]. Therefore, the presence of necrotic teeth in the marginal site of mandibulotomy should be avoided. The aim of this case series was to evaluate whether tooth necrosis is a common event following mandibulectomy or mandibulotomy.

In our study, 15/23 teeth (65.2%) showed a negative response to sensitivity testing. The type of resective surgery did not seem to have any influence on the outcome of pulp sensitivity testing: the rate of nonvital teeth was 66.7% in the mandibulotomy group and 60% in the mandibulectomy group.

In our study, both mesial and distal teeth to mandibulotomy showed a negative response to pulp testing.

Although the small sample size of the included patients did not allow us to perform an inferential statistical analysis, it may be stated that the loss of dental pulp vitality was a frequent finding after mandibulotomy and mandibulectomy. In this study, only two teeth in patients treated with segmental mandibulectomy showed positive cold or electric sensitivity pulp testing.

The hypothesis is that the trauma itself, during surgical intervention and mandibulotomy, can provoke a negative response to sensitivity tests and necrosis of the teeth.

Casey et al. (1999) showed that mandibular teeth mesial to the area of the segmental mandibulectomy can remain vital due to the inferior alveolar artery that supplies dental vessels to the remaining teeth. The retrograde flow in the inferior alveolar artery can occur within a relatively short time and comes from the mental arteries and the inferior alveolar artery from the opposite side or from periosteal vessels, gingival and periodontal ligament vessels [11]. However, the authors concluded that these teeth can be more susceptible to caries and restorative dental procedures that can cause tooth necrosis.

As previously stated, RT is often employed in conjunction with surgery in cases of advanced oral cancers. It is usually a postoperative treatment, and unfortunately, it causes acute and long-term adverse effects [19]. One of the most severe adverse effects is osteoradionecrosis of the jaw (ORN).

The main risk factors for ORN are postirradiation extraction of the mandibular or maxillary teeth within the radiation field, surgical intervention, bacterial infections, total radiation dose, periodontitis, dental status and tumour characteristics [20, 21]. For this reason, it is essential to perform dental preradiation dental screening to eliminate oral foci of infection [22], although pre-RT tooth extraction may also represent a risk factor for ORN [23, 24], while root canal therapy seems to be a safe procedure to be performed, both before and after head and neck radiotherapy, to avoid or delay dental extraction [14].

Nevertheless, our results highlight the need for strict cooperation between the ENT surgeon and dental professionals: considering how 9/15 (60%) nonvital teeth needed treatment during a short follow-up period (8 teeth out of 9 were endodontically treated or extracted during the first year of follow-up), we propose to plan the endodontic treatment of teeth neighbouring the line of osteotomy before oncologic surgery. This could lead to several benefits: the outcome of root canal therapy may be impaired in cases of necrotic pulps with apical periodontitis [25]: Rossi-Fedele & Ng, in a systematic review and meta-analysis, reported how root canal therapy is more effective for prevention than resolution of apical periodontitis [26]. Performing endodontic treatment on a vital tooth would reduce the risk of failure. Moreover, after mandibulectomy or mandibulotomy, the opening of the mouth is usually reduced, and the new shape of the floor of the mouth does not allow the positioning of periapical radiography, making it difficult to perform root canal therapy. Furthermore, the opening can also worsen after head and neck radiotherapy [27].

On the other hand, preventive root canal therapy could be an overtreatment, considering that some teeth preserve a positive response to sensitivity testing. Nevertheless, several studies have demonstrated how the endodontic treatment of a vital tooth is highly predictable [28, 29], only slightly impairing the prognosis of the teeth. However, since the prevention of ORN should be a priority for HNC patients and because of the technical and biological challenges of root canal treatment after oncologic surgery, the risk of overtreatment may be considered of secondary importance.

This study had one major limitation. First, the sample size was small due to its design (case series), which made inferential statistical analysis impossible. Furthermore, it was a monocentric study: all the patients were treated by the same group of surgeons, and it was impossible to prove the external validity of our findings. Further studies are needed to improve the evidence about this topic. Another limitation of this study is a substantial heterogeneity in the follow-up period: in fact, the follow-up ranged between 17 days and 7 years. It should be highlighted that most of the vital teeth received a short follow-up, when compared to the total sample. A longer follow-up of these patients would have provided a more accurate estimate of tooth necrosis after oncologic surgery, and it could be speculated that the incidence of tooth necrosis may become even higher. However, the difficulties in completing the follow-up of HNC patients in clinical trials are well-known among researchers, due to the fragile nature of HNC patients and their impaired survival rate [30].

Another limitation of this study is that all the teeth were evaluated after the oncologic surgery. It could be speculated that some of the included teeth were not vital even before the surgery. This eventuality, although possible, is unlikely: none of the included teeth had caries or showed any other clinical signs that might have suggested that necrosis occurred before the surgery.

This study also showed several strengths: the cold pulp testing used in this study is considered the most effective sensitivity test when applied to the tooth by saturating cotton pellets and placing them in the right part of the teeth [17]. The accuracy of electric pulp testers has been debated because of several false-positives. This test detects functional neurons but does not detect pulpal health [31, 32]. However, the electric pulp test has shown greater accuracy than cold testing, mostly in teeth with canal obliteration and teeth of old patients [33]. It has been used as an alternative to cold pulp testing, but it is suggested for use together with cold testing because of its low sensitivity [34].

## Conclusion

If our results are confirmed by further studies, performing root canal therapy before surgery on the teeth adjacent to the surgical site could be an appropriate strategy. Proper planning of the mandibulotomy site performing root canal therapy or the extraction of teeth adjacent to the mandibulotomy might help to avoid complications.

## Data Availability

The data that support the findings of this study are available from the corresponding author, C.R., upon reasonable request.
